# Beyond Cognitive Support: Social Interaction and Affective Experience in AI-Assisted Creative Thinking Among University Students

**DOI:** 10.3390/jintelligence14070151

**Published:** 2026-07-20

**Authors:** Yawen Cheng, Xiaoting Huang

**Affiliations:** 1 School of Education, Central China Normal University, Wuhan 430079, China; chengyw0226@hotmail.com; 2 Graduate School of Education, Peking University, Beijing 100871, China; 3 Institution of Economics of Education, Peking University, Beijing 100871, China

**Keywords:** creative thinking, convergent thinking, divergent thinking, artificial intelligence, undergraduate students

## Abstract

Creative thinking has become an increasingly important competence in education, yet less is known about how it may be supported in human–AI collaboration as large language models (LLMs) are increasingly employed as partners in learning and problem solving. This study examined whether and how cognitive support and social interaction in AI-assisted collaboration were associated with divergent and convergent thinking. A total of 92 undergraduate students completed creative problem-solving tasks with GPT-4.0. Data were collected from task outputs and self-report measures, and the hypothesized associations were tested using structural equation modeling, with indirect effects examined as supplementary analyses. Results showed that in human–AI collaboration, social interaction rather than cognitive support was associated with more positive affective experiences, and this affective pathway was linked specifically to convergent thinking rather than divergent thinking. These findings suggest that associations between AI-assisted collaboration and creative thinking may depend less on perceived cognitive support alone than on the extent to which collaboration is experienced as socially engaging and affectively positive, particularly for convergent thinking.

## 1. Introduction

Creative thinking, widely recognized as an important aspect of human intelligence, has become increasingly important for personal development, technological innovation, and national competitiveness (OECD, 2023). As problem solving becomes more complex, high-level creative thinking is often difficult to achieve through individual effort alone, while collaboration can facilitate creative thinking by providing cognitive resources and interactional processes that support idea development and sustain engagement (Moirano et al., 2020). Moreover, such collaboration increasingly extends beyond human partners to include large language models, which can participate in idea generation, feedback, and iterative exchange, thereby becoming active partners in creative problem-solving tasks.

Prior research has provided evidence that large language models can have a positive effect on students’ creative thinking. Lee and Chung (2024) found that LLMs can provide learners with ideas and perspectives they might not otherwise have considered, while also helping them identify and explore overlooked aspects of a task. Experimental and interview evidence from Essel et al. (2024) similarly suggested that the use of LLMs may enhance students’ creative thinking. Doshi and Hauser (2024) further showed that LLMs can provide diverse initial ideas, thereby reducing cognitive burden during the problem-solving process, particularly for individuals who may struggle with idea generation.

LLMs may contribute to students’ creative thinking in at least two ways. First, they may provide cognitive support by offering prompts, examples, and alternative perspectives that help students expand ideas and reduce fixation (Boussioux et al., 2024). Second, they may provide a form of social interaction by simulating dialogic exchange and responsive feedback during task performance (Brandtzaeg et al., 2022). These two dimensions of AI-assisted collaboration may be relevant for understanding how students experience and benefit from working with AI. However, most existing studies on AI-assisted creative thinking have focused primarily on whether AI improves creative thinking outcomes, rather than on the processes through which such associations may emerge (Koivisto & Grassini, 2023). As a result, it remains unclear how AI-assisted collaboration is related to creative thinking, and whether the association differs between divergent and convergent thinking, two essential components of creative thinking.

One process that may help account for these associations is affective experience during collaboration. The influence of cognitive and social aspects of interaction may depend partly on how the collaboration is subjectively experienced. Cognitive support may be more effective when it is experienced as engaging rather than merely informative, whereas social interaction may be more consequential when it evokes a sense of responsiveness, involvement, or connection. From this perspective, affective experience may help explain how AI-assisted collaboration is associated with creative thinking outcomes. This possibility is consistent with Broaden-and-Build Theory, which proposes that positive affect broadens momentary thought-action repertoires and supports forms of thinking relevant to creative thinking (Fredrickson, 2001).

Moreover, the pattern of association may differ between divergent thinking and convergent thinking. Divergent thinking places greater emphasis on generating multiple, varied, and original ideas, whereas convergent thinking requires the evaluation, selection, and refinement of ideas into an appropriate solution, thus AI-assisted collaboration may not contribute to them in the same way. It is therefore important to examine whether affective experience is similarly associated with both outcomes, or whether it is more closely related to one form of thinking than the other. Likewise, cognitive support and social interaction may also differ in the extent to which they are associated with students’ affective experience during human–AI collaboration.

To this end, the present study attempts to investigate how LLMs may support students’ creative thinking through cognitive support, social interaction, and affective experience. Using data from 92 undergraduate students who completed creative problem-solving tasks with GPT 4.0, we tested a model linking cognitive support, social interaction, affective experience, and two creative thinking outcomes: divergent thinking and convergent thinking. The study aims to address three research questions:

**RQ1:**  To what extent do cognitive support and social interaction relate to students’ affective experience during AI-assisted collaboration?
**RQ2:**  To what extent does affective experience account for the associations of cognitive support and social interaction with creative thinking outcomes during AI-assisted collaboration?
**RQ3:**  Do these associations differ between divergent thinking and convergent thinking?

## 2. Literature Review

### 2.1. Creative Thinking as Phase-Sensitive Outcomes in AI-Assisted Problem Solving

Creative thinking is commonly defined in terms of two core criteria, novelty and usefulness, and these criteria are often reflected in two complementary but distinguishable forms of thinking: divergent thinking and convergent thinking (Runco & Jaeger, 2012). Divergent thinking refers to the generation of multiple, varied, and original ideas, whereas convergent thinking refers to the evaluation, selection, and integration of ideas into an appropriate response (Guilford, 1967; Cropley, 2006; Nijstad et al., 2010; Zhu et al., 2019). In AI-assisted problem solving, this distinction is particularly important because the two forms of thinking correspond to different functional demands within the creative process. Accordingly, AI-assisted collaboration may not be associated with divergent and convergent thinking in the same way. In the present study, we therefore treat them as phase-sensitive creative outcomes rather than as interchangeable indicators of general creative performance.

### 2.2. Human–AI Collaboration as Perceived Collaborative Support

LLMs have increasingly entered creative thinking contexts as collaborative systems, yet empirical findings on AI-assisted creative thinking remain mixed. Recent human–AI co-creativity research suggests that AI systems may contribute to creative work not only by generating content, but also through interactional processes such as turn-taking, communication, feedback, and iterative refinement (Rezwana & Maher, 2023; Wan et al., 2024). Some studies suggest that generative AI can enhance creative performance by increasing idea production, originality, or flexibility (Lee & Chung, 2024). Other studies have reported less positive findings. Niloy et al. (2024), for instance, found a decline in students’ creative writing performance after ChatGPT 3.5 use, whereas Habib et al. (2024) suggested that generative AI may negatively affect confidence in one’s own creative ability. Meanwhile, related work has also shown that AI does not necessarily outperform humans in divergent-thinking tasks and may sometimes constrain originality by encouraging reliance on familiar or statistically likely outputs (Koivisto & Grassini, 2023).

These mixed findings suggest that the role of AI in creative thinking cannot be understood adequately through outcome comparisons alone. Prior research has focused mainly on whether AI improves creative performance, with less attention to the processes through which students perceive and experience AI support during creative problem solving. However, different features of the interaction may be perceived by students as different forms of collaborative support, and these forms of support may relate differently to creative thinking outcomes.

In the present study, human–AI collaboration is therefore conceptualized as students’ perceived collaborative support during creative problem solving. This support includes two theoretically distinct dimensions: cognitive support and social interaction. Drawing on scaffolding theory, cognitive support refers to task-oriented assistance that enables learners to engage in problem solving beyond their unaided level (Belland, 2014). This view is also consistent with the notion of technologies as partners in cognition, in which cognitive enhancement may occur while individuals work with intelligent tools (Salomon et al., 1991). Recent work in higher education similarly suggests that generative AI is increasingly used to support feedback provision, task guidance, and cognitive scaffolding, particularly when AI agents are combined with structured scaffolding questions (Tillmanns et al., 2025; Yan et al., 2025). In the present study, cognitive support refers specifically to students’ perceived ideational support from the AI agent, including support for broadening perspectives, considering alternative approaches, and exploring unconventional ideas.

Social interaction, by contrast, refers to students’ perceived dialogic and co-constructive quality of interaction with the AI agent. It is grounded in social presence theory, which emphasizes the perceived salience of the other in mediated communication (Short et al., 1976), and in research showing that people may apply social rules and expectations to computers (Nass & Moon, 2000). It is also consistent with work on text-based inquiry environments, where social presence can support collaborative meaning-making (Garrison et al., 1999). Recent work on customized generative AI chatbots further supports this view by showing how AI can be designed as a dialogic partner that promotes multiple perspectives, reasoning, and argumentation rather than functioning only as an authoritative information provider (Tang & Putra, 2026). In the present context, social interaction refers to the extent to which the AI agent is experienced as responsive, dialogic, and jointly exploratory. Taken together, cognitive support and social interaction represent two distinct dimensions of perceived AI support: the former concerns students’ experience of ideational assistance, whereas the latter concerns the co-constructive support of the interaction.

### 2.3. Affective Experience as the Proximal Process

The distinction between cognitive support and social interaction raises a further question: through what proximal process might these forms of perceived AI support be associated with creative thinking? In this study, affective experience is positioned as such a theoretically proximal process that refers to task-specific motivational and emotional engagement during AI-assisted creative problem solving, reflected in students’ motivation, enjoyment, and interest in the task. This conceptualization is consistent with the intrinsic motivation principle of creativity, which emphasizes the importance of intrinsic motivation for creative work (Amabile, 2013), and with control-value theory, which explains how task-related emotions such as enjoyment and interest are connected to motivation and engagement in academic contexts (Pekrun, 2006).

Cognitive support and social interaction may be associated with affective experience through different theoretical routes. From the perspective of self-determination theory, supportive contexts are more likely to foster positive motivational and affective functioning when they support competence, autonomy, and relatedness (Ryan & Deci, 2000). Cognitive support may contribute primarily to perceived competence by helping students organize ideas, consider alternatives, and move beyond initial responses. In AI-assisted creative tasks, such ideational assistance may reduce uncertainty and make idea development feel more manageable. Social interaction, by contrast, may contribute more directly to perceived relatedness by making the collaboration feel responsive, dialogic, and jointly exploratory. This argument is also supported by social presence theory and research on social responses to computers, which suggest that users may experience mediated or computer-based interaction as socially salient even when the system does not possess genuine social agency (Nass & Moon, 2000). Related work on text-based inquiry environments and conversational agents further suggests that perceived social presence and socially responsive cues can support collaborative meaning-making, emotional connection, and relational interpretations of interaction (Garrison et al., 1999; Brandtzaeg et al., 2022).

Affective experience is also relevant to creative thinking outcomes. Positive affect has been shown to facilitate creative problem solving (Isen et al., 1987), and broaden-and-build theory suggests that positive affect can broaden thought-action repertoires and support flexible cognition (Fredrickson, 2001). At the same time, mood-creativity research indicates that the role of affect depends on task demands rather than operating uniformly across all forms of creativity (Baas et al., 2008; Nijstad et al., 2010). This task-dependent perspective indicates that divergent and convergent thinking may involve different creative demands, for which the present study examines whether affective experience shows similar or distinct associations with divergent and convergent thinking.

### 2.4. The Proposed Process Model

Prior findings on generative AI and creative thinking remain mixed, suggesting that the role of AI may depend on the processes through which students perceive and experience AI support. Building on the preceding review, we propose a process model of AI-assisted creative thinking. In this model, perceived AI support is differentiated into cognitive and socio-interactional dimensions, affective experience is treated as a proximal motivational-affective process, and divergent and convergent thinking are examined as distinct creative outcomes. This integrated account allows us to move beyond the question of whether AI improves creative thinking and to examine how different perceived qualities of AI collaboration may be associated with different forms of creative thinking. Given the correlational and process-oriented nature of the present study, these hypotheses concern directional associations among perceived AI support, affective experience, and creative thinking outcomes rather than causal effects.

**H1a.**  *Perceived cognitive support is positively associated with students* *’ affective experience during AI-assisted creative thinking.*

**H1b.**  *Perceived social interaction is positively associated with students* *’ affective experience during AI-assisted creative thinking.*

**H2a.**  *Perceived cognitive support is indirectly associated with creative thinking outcomes through students* *’ affective experience.*

**H2b.**  *Perceived social interaction is indirectly associated with creative thinking outcomes through students* *’ affective experience.*

**H3.**  *The pattern of associations among perceived AI support, affective experience, and creative thinking outcomes differs between divergent and convergent thinking.*

## 3. Methods

### 3.1. Participants and Sampling

This research was approved by the Institutional Review Board at Peking University. Participants were recruited from University A through a voluntary advertisement for a creativity assessment study. Informed consent was obtained from all the participants and the participants had the right to not participate in the study. Eligible participants were undergraduate students who were native Chinese speakers, reported being in good physical and psychological condition, and were able to complete the online creative tasks. The final analytic sample included participants who met these criteria, completed the creative-thinking tasks and questionnaires, and passed data-quality screening. Responses were excluded if they were incomplete, completed in an unrealistically short time, or contained obvious invalid or low-quality content. The final sample consisted of 44 females and 48 males, ranging from first- to fifth-year students, including 44 freshmen, 18 sophomores, 9 juniors, and 21 seniors or fifth-year students.^1^

### 3.2. Sample Size and Power Consideration

An a priori SEM power analysis was conducted using semPower to evaluate the sample size required for detecting the focal structural paths in the proposed model. The analysis specified a target structural path of β = 0.30, α = 0.05, desired power of 0.80, and a correlation of 0.20 between the two exogenous latent variables. Under this specified focal-path scenario, the required sample size was 84 participants. The final analytic sample of 92 participants was therefore above the required N for this specified focal-path scenario. However, because indirect associations typically require greater statistical power than single structural paths, the indirect-path findings were interpreted cautiously as suggestive rather than definitive.

### 3.3. Study Design and Procedure

Participants completed three AI-assisted creative tasks online, which covered various fields: daily life issues (the quality of online courses), social issues (concerns related to the decline in fertility rate), and scientific challenges (sustainable development). Each task lasted 10 min, resulting in a total task duration of approximately 30 min. Before beginning the tasks, participants were instructed to log into a designated ChatGPT account using GPT-4.0 and to begin the interaction with a standardized prompt: “Let us work together to complete a series of creative tasks.” Participants were then allowed to interact with GPT-4.0 iteratively, including asking follow-up questions, requesting elaboration, or refining the generated ideas, until they obtained an answer they considered satisfactory. After each task, participants copied or summarized the final answer into the corresponding response box in the online questionnaire. During the task, participants were asked to close unrelated social software and webpages to reduce distraction. The divergent and convergent thinking components were separated by task requirements and scoring procedures: divergent-thinking responses focused on the generation of multiple and original ideas, whereas convergent-thinking responses required participants to evaluate and refine ideas toward a final solution. These responses were submitted in separate fields and scored according to distinct criteria.

### 3.4. Measures

#### 3.4.1. Creative Thinking

As noted above, creative thinking was defined as the capacity to generate ideas that are both novel and useful in AI-assisted problem solving, integrating both divergent and convergent thinking processes in the present study. Accordingly, each task was divided into two phases: a divergent thinking phase, where human–AI collaboration generated as many relevant solutions as possible based on the problem context; and a convergent thinking phase, where human–AI collaboration evaluated, analyzed, and refined their initial ideas to determine a final solution. Scoring is based on students’ submitted final responses. The assessment items demonstrated high internal consistency (Cronbach’s α = 0.864).

Scoring utilized a hybrid approach combining LLM automation with expert supervision. For divergent thinking, performance was evaluated in terms of fluency, flexibility, and originality. Fluency refers to the number of valid ideas, flexibility to the number of distinct ideas, and originality to the number of uncommon ideas. However, these indicators are often highly correlated, particularly because flexibility and originality are strongly influenced by fluency, which has raised concerns about the validity of divergent thinking scores. In addition, prior research has noted that raw scores may introduce scoring bias if they are not adjusted for fluency. To address this issue, we followed Forthmann et al. (2020), who recommended ratio-based quality scores as the default approach for scoring divergent thinking. Accordingly, divergent thinking was operationalized as the sum of the flexibility-to-fluency ratio and the originality-to-fluency ratio. DeepSeek-R1 was prompted to score students’ responses according to these standardized criteria in order to improve scoring objectivity and consistency.

For convergent thinking, DeepSeek-R1 evaluated the utility of students’ improvement plans in light of the response context. The scoring prompt was designed as a rule-based and standardized framework to enhance consistency, transparency, and interpretability. Feasibility was assessed across three dimensions—time cycle, budget feasibility, and optimization efficiency—using clearly defined five-point criteria, and the final score was calculated as the average of the three dimension scores (1 = absolutely not feasible; 5 = very feasible). The scoring AI was also required to follow the scoring rules strictly and to provide a brief justification for each score. This design helped ensure that AI-based scoring remained structured, evidence-based, and comparable across responses. For each of the three creative-thinking tasks, the dimension scores were standardized before being combined into the overall divergent-thinking or convergent-thinking score.

GPT-4.0 was used as the collaborative agent, whereas DeepSeek-R1 was used here only for scoring. This separation was aimed at reducing potential same-model evaluation bias, which has been identified as a concern in recent LLM-as-a-judge research (Panickssery et al., 2024). The scoring was guided by pre-specified prompts, rubrics, decision rules, and standardized output requirements; the complete prompts and rubrics are provided in the Appendix D. To validate the AI-assisted scoring procedure, two trained human evaluators with experience in creativity assessment independently scored a randomly selected 20% of participant-task responses after reviewing the rubrics and completing a calibration discussion. All responses were anonymized, and the evaluators were blind to participants’ identities, questionnaire responses, model variables, and DeepSeek-R1 scores during independent scoring. Disagreements between the two evaluators were resolved through discussion to form consensus ratings, which were then compared with the DeepSeek-R1 scores. The AI-assisted scores showed high agreement with the consensus human ratings, as indicated by an exact agreement rate of 91.67%, a Pearson correlation coefficient of 0.983, a linearly weighted Cohen’s kappa of 0.956, and an intraclass correlation coefficient for absolute agreement (ICC[A,1]) of 0.983.

#### 3.4.2. Cognitive Support, Social Interaction, and Affective Experience

The self-report measures of cognitive support, social interaction, and affective experience were developed with reference to Lee and Chung (2024), which provided a validated basis for assessing students’ perceived experiences in AI-assisted creative tasks. For the present study, the items were adapted to the human–AI collaboration context by anchoring them in participants’ experience of completing creative problem-solving tasks with ChatGPT.

Cognitive support was conceptualized as students’ perceived AI-enabled cognitive support during collaborative problem solving in this study. Specifically, it referred to participants’ subjective perceptions of the extent to which the AI agent supported key cognitive processes involved in idea development, such as broadening perspectives, stimulating further thinking, and facilitating exploration of possible directions. Participants rated three items on a five-point Likert scale (1 = strongly disagree, 5 = strongly agree), assessing the extent to which the AI agent helped them (a) explore alternative perspectives, (b) expand their thinking about the task, and (c) consider a wider range of possible ideas. The scale showed acceptable internal consistency (Cronbach’s α = 0.776).

Social interaction was conceptualized as students’ perceived interactive and co-constructive quality of collaboration with the AI agent. It reflected participants’ subjective perceptions of the extent to which the interaction with the AI agent was dialogic, responsive, and collaboratively oriented during task completion. Participants rated three items on a five-point Likert scale (1 = strongly disagree, 5 = strongly agree), which assessed dialogic and collaborative interaction, including whether participants felt that they were (a) engaged in a conversation, (b) brainstorming together, and (c) jointly exploring ideas with the AI agent. The scale demonstrated good internal consistency (Cronbach’s α = 0.804).

Affective experience was conceptualized as students’ subjective motivational and emotional engagement during AI-assisted collaborative problem solving. It reflected the extent to which participants experienced the task as engaging, enjoyable, and interesting. Three items were rated on seven-point scales (1 = not at all, 7 = very much), assessing participants’ motivation, liking, and interest during the task. The scale also showed acceptable internal consistency (Cronbach’s α = 0.785). Appendix A provides a detailed description of the items in each questionnaire.

### 3.5. Data Analysis

All analyses were conducted in Stata 16. The final analytic sample consisted of complete cases after data-quality screening; therefore, no missing data handling or imputation was required.

Specifically, descriptive statistics and Pearson correlation coefficients were first computed for all study variables, including cognitive support, social interaction, affective experience, divergent thinking, convergent thinking, as well as students’ gender and age.

**Figure 1:**
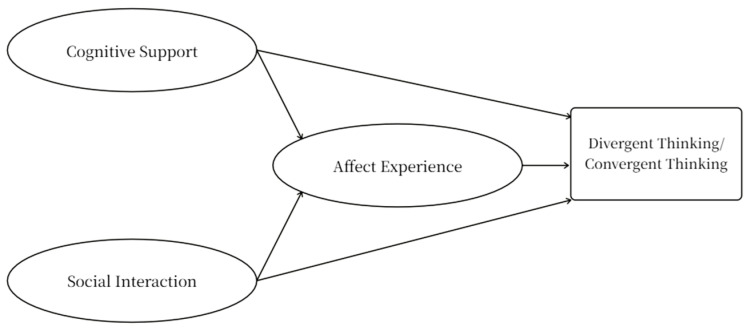
Theoretical model.

Second, the study distinguished between divergent thinking and convergent thinking; thus, two outcomes were examined separately throughout the analyses. Following Iacobucci et al. (2007), mediation effects were examined within a structural equation modeling (SEM) framework rather than through a series of separate regression equations. This approach allows direct and indirect effects to be estimated simultaneously and is particularly appropriate for models involving latent constructs measured by multi-item scales. Accordingly, two SEMs were estimated: one with divergent thinking as the outcome and one with convergent thinking as the outcome. In both models, cognitive support and social interaction were specified as exogenous perceived support variables, affective experience as a process variable, and gender and age as control variables. Structural equation models were estimated using maximum likelihood estimation and standardized coefficients were reported to facilitate interpretation. Figure 1 shows the theoretical model of the study.

To further assess indirect effects, mediation analysis was conducted in Stata using the medsem package (Mehmetoglu, 2018). Indirect effects were computed as the product of the relevant path coefficients. Specifically, bootstrap analyses with 1000 resamples were conducted to obtain bias-corrected 95% confidence intervals for the indirect effects. Sobel, delta-method, and Monte Carlo estimates were also reported as supplementary checks to assess the consistency of the indirect associations across different estimation approaches. However, given the cross-sectional design and modest sample size, the mediation-related analyses were interpreted as evidence of indirect statistical associations rather than causal mediation.

Model fit was evaluated using the comparative fit index (CFI), Tucker–Lewis index (TLI), standardized root mean square residual (SRMR), and root mean square error of approximation (RMSEA). The CFI is relatively more biased toward determining model fit independent of sample size, and the qualification standard for CFI is 0.9 (McDonald & Ho, 2002). Sharma et al. (2005) concluded that the performance of the TLI test model fit is the best among all the groups of test metrics, with a suggested threshold criterion of 0.9. A rule of thumb is that the SRMR should be less than 0.05 for a good fit (Hu & Bentler, 1999), whereas values smaller than 0.10 may be interpreted as acceptable (Schermelleh-Engel et al., 2003). According to Browne and Cudeck (1992), RMSEA values ≤ 0.05 can be considered as a good fit, values between 0.05 and 0.08 as an adequate fit, and values between 0.08 and 0.10 as a mediocre fit, whereas values > 0.10 are not acceptable.

## 4. Results

### 4.1. Descriptive Statistics and Correlations

Table 1 presents the descriptive statistics and correlations among the study variables. Divergent thinking and convergent thinking were not significantly correlated, supporting their treatment as distinct outcomes. Social interaction was positively associated with affective experience, whereas cognitive support was not significantly related to affective experience. Affective experience was positively associated with convergent thinking but not with divergent thinking. This selective correlation pattern suggests that affective experience captured task-specific motivational and emotional engagement rather than a uniformly positive evaluation of AI-assisted collaboration, providing preliminary descriptive evidence for subsequent testing.

### 4.2. Common Method Bias Test

To assess the potential influence of common method bias, Harman’s single-factor test and multicollinearity diagnostics were conducted (Podsakoff et al., 2003). The first factor accounted for 30.7% of the total variance, which was below the conventional threshold of 40%. In addition, all variance inflation factor values were below 5, with a mean VIF of 1.85. These results suggest that common method bias was unlikely to fully account for the observed associations.

As an additional check for common method bias, we estimated SEMs with a single unmeasured latent method factor loading equally on all self-reported indicators of cognitive support, social interaction, and affective experience. The method factor was constrained to be orthogonal to the substantive latent constructs and covariates. The substantive pattern of results remained broadly consistent after controlling for this constrained method factor, suggesting that the main findings were unlikely to be fully attributable to a single common method factor. Detailed results are reported in the Appendix C.

### 4.3. The Relationship Between Cognitive Support, Social Interaction, Affective Experience and Divergent Thinking

Table 2 presents the structural model with divergent thinking as the outcome. The model showed acceptable fit to the data (CFI = 0.990, TLI = 0.985, SRMR = 0.063, RMSEA = 0.026). Social interaction was positively associated with affective experience (β = 0.362, *p* = 0.002), whereas cognitive support was not significantly associated with affective experience (β = −0.144, *p* = 0.239). In substantive terms, the standardized coefficient for social interaction indicates a moderate positive association with affective experience: students who perceived the AI interaction as more dialogic and co-constructive tended to report more positive motivational and emotional engagement during human–AI collaboration.

In addition, affective experience was not significantly associated with divergent thinking (β = 0.090, *p* = 0.466), and the size of this coefficient was weak. Also, the direct paths from the two perceived support dimensions to divergent thinking were not statistically significant. The point estimate for cognitive support was close to zero (β = −0.018, *p* = 0.875), while the estimate for social interaction was also negative (β = −0.231, *p* = 0.059). Overall, these findings do not support an affective process to divergent thinking. Although social interaction was meaningfully related to affective experience, this positive experience did not appear to translate into stronger divergent-thinking performance in the present sample.

### 4.4. The Relationship Between Cognitive Support, Social Interaction, Affective Experience and Convergent Thinking

Table 3 presents the structural model with convergent thinking as the outcome. The model showed acceptable fit to the data (CFI = 0.994, TLI = 0.991, SRMR = 0.062, RMSEA = 0.020). As in the divergent-thinking model, social interaction was positively associated with affective experience (β = 0.373, *p* = 0.001), whereas cognitive support was not significantly associated with affective experience (β = −0.159, *p* = 0.192). The standardized coefficient for social interaction indicates a moderate association, suggesting that the perceived dialogic and co-constructive quality of AI interaction was substantively related to students’ affective experience.

Meanwhile, affective experience was positively associated with convergent thinking (β = 0.336, *p* = 0.008). This coefficient reflects a moderate association, indicating that students who reported more positive affective experience tended to perform better in the convergent phase of creative thinking. By contrast, the direct paths from cognitive support to convergent thinking (β = 0.182, *p* = 0.105) and from social interaction to convergent thinking (β = −0.132, *p* = 0.301) were not statistically significant. The above results suggest that perceived social interaction was related to convergent thinking mainly through affective experience rather than through a direct association with performance.

### 4.5. Indirect Effect Analysis

Table 4 further reports supplementary analyses of the indirect association between social interaction and convergent thinking through affective experience. The bias-corrected bootstrap confidence interval for the indirect effect did not include zero, suggesting a possible indirect statistical association. Similar patterns were observed for the Sobel and Monte Carlo estimates, whereas the delta-method confidence interval slightly included zero. Taken together, these results provide cautious and suggestive evidence for an indirect pathway from social interaction to convergent thinking through affective experience.

## 5. Discussion

This study examined whether cognitive support and social interaction in AI-assisted collaboration were associated with divergent and convergent thinking through affective experience. Three findings are particularly noteworthy. First, social interaction, but not cognitive support, was consistently associated with affective experience. Second, affective experience was positively associated with convergent thinking but not with divergent thinking. Third, the results provided suggestive evidence for an indirect pathway from social interaction to convergent thinking through affective experience. Taken together, these findings point to a selective rather than general pattern of association linking perceived human–AI collaboration, affective experience, and creative thinking.

A first implication is that the socially experienced quality of human–AI collaboration may be relevant to students’ affective engagement during creative problem solving. In the present study, students who experienced the AI interaction as more responsive, dialogic, and jointly exploratory reported more positive affective experience. This finding suggests that, in educational settings, the design of AI-assisted creative tasks should not focus only on the informational quality of AI outputs. It may also be important to guide students to interact with AI in ways that promote dialogue, questioning, and co-construction, such as asking the AI to compare alternatives, challenge assumptions, explain its suggestions, or help refine an emerging solution. This interpretation is consistent with social-constructivist perspectives, which emphasize that higher-order thinking is shaped through socially mediated interaction rather than information delivery alone, as well as with human-chatbot research showing that conversational agents can elicit perceptions of social presence and relational engagement under some conditions (Vygotsky, 1978; Skjuve et al., 2022).

A second implication is that affective experience was associated with convergent thinking but not divergent thinking in the present model. This pattern suggests that positive motivational and emotional engagement in AI-assisted collaboration may be more relevant when students need to evaluate, integrate, and refine ideas than when they mainly need to generate multiple and original possibilities. Convergent thinking requires learners to compare alternatives and integrate solutions with their own judgment to reach an appropriate solution (Cropley, 2006). By contrast, divergent thinking depends more directly on producing varied and original ideas, and positive task engagement alone may not be sufficient to ensure such ideational diversity. This distinction is particularly important in AI-assisted contexts, where generative AI may sometimes anchor users’ thinking, encourage convergence around similar outputs, or fail to enhance divergent ideation in a straightforward way (Koivisto & Grassini, 2023; Boussioux et al., 2024; Doshi & Hauser, 2024; McGuire et al., 2024). Practically, the observed pattern points to phase-specific uses of AI support. Socially engaging and positive interaction may be most useful when students refine ideas, for example by justifying a selected option, comparing alternatives, and revising a solution through dialogue. For divergent thinking, positive engagement may need to be paired with ideational scaffolds that prompt unusual perspectives, cross-category alternatives, and deliberate movement beyond the AI’s initial suggestions. This interpretation aligns with evidence that affect-creativity relations vary by task demands rather than operating as a uniform facilitative process (Baas et al., 2008; Nijstad et al., 2010).

The non-significant findings for cognitive support do not rule out its value, but should be interpreted cautiously. One possible implication is that simply providing ideas, examples, or alternative directions may be insufficient unless students are also supported in using these inputs actively and reflectively. In educational applications, teachers may therefore need to structure how students work with AI-generated suggestions, for example by requiring them to compare AI responses, explain why a suggestion is useful, adapt it to the task constraints, or combine it with their own reasoning. Taken together, the findings caution against treating AI support as a unitary instructional resource. They suggest that AI may need to be used differently across creative phases: more targeted scaffolding may be needed for divergent idea generation, whereas socially engaging and affectively supportive interaction may be especially relevant for convergent evaluation and refinement.

Overall, this study contributes to the literature in three respects. First, it distinguishes cognitive support from social interaction as two theoretically different dimensions of perceived support in human–AI collaboration. Second, it suggests that affective experience may represent a selective process variable rather than a universal explanatory pathway, with stronger relevance for convergent than for divergent thinking. Third, it suggests that the socially experienced quality of interaction with AI may be more closely related to affective engagement and refinement-oriented creative performance than instrumental cognitive support alone. In this sense, the findings refine broader claims that AI generally enhances creative thinking by showing that its contribution may depend on both the type of support provided and the type of creative thinking considered.

Several limitations point to specific directions for future research. First, the modest sample and cross-sectional design reduce the stability of parameter estimates, especially for indirect associations. Experimental and longitudinal studies are needed to test the proposed process. Researchers could manipulate AI dialogic responsiveness while holding informational quality constant and examine whether perceived social presence and relatedness satisfaction explain its effect on task-specific affective experience, referring to social presence theory and self-determination theory (Short et al., 1976; Ryan & Deci, 2000). Second, the association of affective experience with convergent but not divergent thinking requires phase-specific investigation. Affect-creativity relationships depend on task demands and the need for cognitive flexibility or persistence (Baas et al., 2008; Nijstad et al., 2010), thus future studies could manipulate AI responsiveness separately during divergent and convergent phases and assess affective experience and performance after each phase. Third, the non-significant findings for cognitive support suggest that access to AI-generated ideas should be distinguished from their active uptake. Scaffolding theory indicates that such support may be more relevant when students evaluate, adapt, or integrate AI suggestions rather than accept them passively (Belland, 2014). This proposition could be tested by comparing suggestion-only conditions with conditions that require students to compare alternatives, justify their choices, or revise AI suggestions. Interaction logs and repeated process measures could further test the proposed sequence among perceived support, active uptake, and affective experience. Finally, the AI-assisted scoring procedure and single-university convenience sample may limit the robustness and generalizability of the findings. Future studies could test the proposed model using multisite samples and assess the robustness of the results through alternative AI scoring models.

## 6. Conclusions

AI-assisted collaboration did not relate to creative thinking in a uniform way. In this study, perceived social interaction, rather than cognitive support, was associated with more positive affective experience, and affective experience was associated only with convergent thinking. In addition, an indirect pathway appeared from social interaction to convergent thinking through affective experience. Overall, the findings suggest that the observed association between AI-assisted collaboration and creative thinking may depend less on perceived cognitive help alone than on whether the collaboration is experienced as socially engaging and affectively positive, particularly during the evaluation and refinement of ideas.

## Figures and Tables

**Table 1:** Descriptive statistics and correlations between the investigated variables.

	Mean	SD	1	2	3	4	5	6	7
Divergent Thinking (1)	−2.915	2.546	-						
Convergent Thinking (2)	0.759	1.683	0.026	-					
Cognitive Support (3)	4.123	0.646	−0.016	0.130	-				
Social Interaction (4)	3.551	0.851	−0.206 *	−0.011	0.093	-			
Affective Experience (5)	5.058	0.951	0.003	0.263 *	−0.083	0.275 *	-		
Male (6)	0.522	0.502	−0.100	0.018	0.071	0.143	0.097	-	
Age (7)	20.348	1.515	−0.191	−0.077	−0.070	0.052	−0.154	−0.169	-

Note: * *p* < 0.05.

**Table 2:** The relationship between cognition, social, affect and divergent thinking.

Type	Effect	Estimate (β)	SE	95% CI		*p*
X_1_ → Y	Cognitive Support → Divergent Thinking	−0.018	0.113	−0.239	0.203	0.875
X_2_ → Y	Social Interaction → Divergent Thinking	−0.231	0.122	−0.471	0.009	0.059
M → Y	Affective Experience → Divergent Thinking	0.090	0.124	−0.152	0.333	0.466
X_1_ → M	Cognitive Support → Affective Experience	−0.144	0.122	−0.383	0.095	0.239
X_2_ → M	Social Interaction → Affective Experience	0.362	0.117	0.132	0.593	0.002 *

Note: X_1_ = cognitive support; X_2_ = social interaction; M = affective experience; Y = divergent thinking. Standardized coefficients are reported. * *p* < 0.05.

**Table 3:** The relationship between cognition, social, affect and convergent thinking.

Type	Effect	Estimate (β)	SE	95% CI		*p*
X_1_ → Y	Cognitive Support → Convergent Thinking	0.182	0.112	−0.038	0.402	0.105
X_2_ → Y	Social Interaction → Convergent Thinking	−0.132	0.128	−0.384	0.119	0.301
M → Y	Affective Experience → Convergent Thinking	0.336	0.127	0.086	0.585	0.008 *
X_1_ → M	Cognitive Support → Affective Experience	−0.159	0.122	−0.398	0.080	0.192
X_2_ → M	Social Interaction → Affective Experience	0.373	0.117	0.144	0.602	0.001 *

Note: X_1_ = cognitive support; X_2_ = social interaction; M = affective experience; Y = convergent thinking. Standardized coefficients are reported. * *p* < 0.05.

**Table 4:** The indirect effect of social interaction on convergent thinking.

Estimates	Indirect Effect	SE	95% CI
Bootstrap	0.179	0.112	0.023, 0.503
Sobel	0.125	0.062	0.005, 0.246
Delta	0.125	0.066	−0.004, 0.254
Monte Carlo	0.133	0.075	0.024, 0.302

Note: Indirect effects were evaluated primarily using 95% confidence intervals, because the sampling distribution of indirect effects is often asymmetric. Confidence intervals that do not include zero suggest evidence of an indirect statistical association. Given the cross-sectional design and modest sample size, these estimates should be interpreted as suggestive rather than definitive evidence of mediation.

## Data Availability

Data will be available upon reasonable request from the corresponding author.

## Note

1 As a supplementary robustness check addressing the overrepresentation of first-year students, we re-estimated the SEMs by adding a dummy variable for first-year status as an additional control variable. The substantive pattern of results remained unchanged, and the results are reported in the Appendix B.

## Appendix A. Instruments for Cognitive Support, Social Interaction and Affective Experience

**Cognitive Support (Self-reported 5-point Likert Scale) Cronbach’s α = 0.776**
Working with my team/ChatGPT prompted me to consider unique perspectives that I would not typically think of.
Working with my team/ChatGPT prompted me to try novel approaches that I would not typically use.
Working with my team/ChatGPT helped me explore more unconventional creative ideas.
**Social Interaction (Self-reported 5-point Likert Scale) Cronbach’s α = 0.804**
I felt that I was engaging in a dialogue with my team/ChatGPT.
I felt that I was brainstorming with my team/ChatGPT.
I felt that I was socially interacting with my team/ChatGPT to jointly discuss and develop ideas.
**Affective Experience (Self-reported 7-point Likert Scale) Cronbach’s α = 0.785**
How motivated were you to complete this task?
How much did you enjoy participating in this task?
How interesting did you find this task?

## Appendix B. The Relationship Between Cognition, Social, Affect and Creative Thinking with First-Year-Student Dummy Controlled

**Type**	**Effect**	**Estimate (β)**	**SE**	**95% CI**		***p***
X_1_ → Y	Cognitive Support → Divergent Thinking	0.026	0.114	−0.196	0.249	0.816
X_2_ → Y	Social Interaction → Divergent Thinking	−0.234	0.122	−0.473	0.005	0.055
M → Y	Affective Experience → Divergent Thinking	0.070	0.127	−0.180	0.320	0.581
X_1_ → M	Cognitive Support → Affective Experience	−0.083	0.123	−0.324	0.158	0.498
X_2_ → M	Social Interaction → Affective Experience	0.358	0.115	0.132	0.585	0.002 *
Note: X_1_ = cognitive support; X_2_ = social interaction; M = affective experience; Y = divergent thinking. Standardized coefficients are reported. * *p* < 0.05.						

**Type**	**Effect**	**Estimate (β)**	**SE**	**95% CI**		***p***
X_1_ → Y	Cognitive Support → Convergent Thinking	0.182	0.112	−0.039	0.402	0.106
X_2_ → Y	Social Interaction → Convergent Thinking	−0.134	0.128	−0.385	0.117	0.295
M → Y	Affective Experience → Convergent Thinking	0.338	0.131	0.081	0.595	0.010 *
X_1_ → M	Cognitive Support → Affective Experience	−0.098	0.123	−0.339	0.143	0.425
X_2_ → M	Social Interaction → Affective Experience	0.368	0.115	0.143	0.593	0.001 *
Note: X_1_ = cognitive support; X_2_ = social interaction; M = affective experience; Y = convergent thinking. Standardized coefficients are reported. * *p* < 0.05.						

## Appendix C. The Relationship Between Cognition, Social, Affect and Creative Thinking with Common Method Bias Controlled

**Type**	**Effect**	**Estimate (β)**	**SE**	**95% CI**		***p***
X_1_ → Y	Cognitive Support→ Divergent Thinking	0.022	0.110	−0.194	0.238	0.843
X_2_ → Y	Social Interaction → Divergent Thinking	−0.230	0.122	−0.469	0.008	0.059
M → Y	Affective Experience → Divergent Thinking	0.086	0.124	−0.157	0.330	0.487
X_1_ → M	Cognitive Support → Affective Experience	0.157	0.123	−0.085	0.399	0.203
X_2_ → M	Social Interaction → Affective Experience	0.350	0.119	0.115	0.584	0.003 *
Note: X_1_ = cognitive support; X_2_ = social interaction; M = affective experience; Y = divergent thinking. Standardized coefficients are reported. * *p* < 0.05.						

**Type**	**Effect**	**Estimate (β)**	**SE**	**95% CI**		***p***
X_1_ → Y	Cognitive Support → Convergent Thinking	0.214	0.121	−0.024	0.452	0.078
X_2_ → Y	Social Interaction → Convergent Thinking	−0.107	0.127	−0.356	0.142	0.399
M → Y	Affective Experience → Convergent Thinking	0.364	0.134	0.102	0.626	0.006 *
X_1_ → M	Cognitive Support → Affective Experience	−0.238	0.130	−0.492	0.017	0.068
X_2_ → M	Social Interaction → Affective Experience	0.300	0.130	0.045	0.555	0.021 *
Note: X_1_ = cognitive support; X_2_ = social interaction; M = affective experience; Y = convergent thinking. Standardized coefficients are reported. * *p* < 0.05.						

## Appendix D. AI-Assisted Scoring Prompts

**Prompts for scoring divergent thinking:**
“As an expert in evaluating divergent thinking, you must strictly follow the following rules when scoring the students’ responses.
Phase 1: Content Verification
Check if the student’s answer is a creative idea relevant to the task:
→ Yes: Proceed to the [Detailed Scoring Mode]
→ No: Enter the [Invalid Scoring Mode]
Phase 2: Mode Execution
[Invalid Scoring Mode]
If the student’s answer is blank, irrelevant, or merely repeats the requirements of the question:
Fluency = 0
Flexibility = 0
Originality = 0
Only return the judgment mode and the three scores.
[Detailed Scoring Mode]
Please score strictly based on the student’s original response.

1. Fluency
  Fluency refers to the number of effective and non-repetitive ideas presented by the student in the given problem situation.
  - Divide the response according to punctuation marks, numbers, line breaks, and semantic boundaries. Each semantically independent idea is regarded as a potential response.
  - Exclude irrelevant, vague, incomprehensible, merely repeating the question, or responses with no substantive content.
  - If multiple responses express the same core meaning, only one is retained.
2. Flexibility
  Flexibility refers to the number of different idea categories that students employ in their responses to a given problem situation.
  - Classify each valid idea according to the category and key words. The key words are merely for assistance; the final classification is based on semantic meaning.
  - Each valid idea can only be classified into one main category.
  - If a valid idea cannot be classified into any existing category, and if it has a clear semantic meaning, is relevant to the task, and has a substantial difference from the existing categories, it can be classified into a new category.
3. Originality
  Originality refers to the rarity of the idea categories in students’ responses within a given problem context in the entire sample response pool.
  - Calculate based on the category classifications from the flexibility scoring stage. For each category, calculate its frequency proportion in the entire sample response pool.
  - Convert this proportion into a rarity weight: Originality weight = 1 − frequency proportion
  - Less frequent categories therefore receive higher originality weights.
  - The student’s originality score is the sum of the rarity weights of all distinct categories represented in the student’s valid ideas.

Output requirements for the results:

① The determination mode must be declared before the scoring.
② The final score must strictly correspond to the three dimensions of fluency, flexibility and originality.
③ Only output: determination mode, fluency, flexibility, originality.
④ No additional explanations are needed unless the answer is invalid.
⑤ If an explanation is required, it should be within 50 words.

Student’s response:”
**Prompts for scoring convergent thinking:**
“As an expert in evaluating convergent thinking, you must strictly follow the following decision tree structure to conduct a structured assessment:
Phase 1: Content Verification (Mandatory Binary Judgment)
Check whether the student’s answer involves specific time schedules, budget allocations, or optimization strategies (either one is sufficient):
→ Yes: Proceed to the [Detailed Scoring Mode]
→ No: Proceed to the [Theoretical Derivation Mode]
Phase 2: Mode Execution
Based strictly on the determination made in the previous stage, proceed to either the detailed scoring mode or the theoretical scoring mode. Directly entering the detailed scoring mode is prohibited!
[Detailed Scoring Mode]
When conducting a three-dimensional quantitative assessment, the following conditions must be met:
Time period: 5 = Completely feasible 4 = Minor adjustment required 3 = Adjustment needed 2 = Major adjustment required 1 = Infeasible
Budget: 5 = Fully matched 4 = Slight over-budget 3 = Need for re-planning 2 = Severe over-budget 1 = Infeasible
Optimization efficiency: 5 = Significant improvement 4 = Partial improvement 3 = No improvement 2 = Efficiency decline 1 = Severe waste
→ Calculation formula: (Time score + Budget score + Efficiency score)/3 (rounded to two decimal places)
[Theoretical Derivation Mode]
Based on disciplinary theory: Even if not explicitly mentioned, it is necessary to conduct constructive deductions based on disciplinary theory and industry benchmarks:
Time period: Based on technical characteristics/functional complexity, estimate according to the development cycle of similar projects
Budget: Combine the number of functional modules and technical requirements, estimate based on the cost structure of educational technology projects
Optimization efficiency: Based on the innovation of functions and the maturity of existing technologies, assess the potential improvement space
→ Calculation formula: (Time score + Budget score + Efficiency score)/3 (rounded to two decimal places)

Output requirements for the results:

① Before scoring, the type of determination mode must be declared
② The final score must strictly correspond to the theoretical framework
③ For scores of 2–4, only the numbers need to be returned, no other outputs are required!
④ For 1/5 score, an 50-word assessment basis (key defect/innovative highlight) must be attached

Student’s response:”
